# Quantitative Characterization of Structural and Mechanical Properties of Boron Nitride Nanotubes in High Temperature Environments

**DOI:** 10.1038/s41598-017-11795-9

**Published:** 2017-09-12

**Authors:** Xiaoming Chen, Christopher M. Dmuchowski, Cheol Park, Catharine C. Fay, Changhong Ke

**Affiliations:** 1 Micro- and Nanotechnology Research Center, State Key Laboratory for Manufacturing Systems Engineering, Xi’an Jiaotong University, Xi’an, Shaanxi 710049 China; 2 Department of Mechanical Engineering, State University of New York at Binghamton, Binghamton, New York 13902 USA; 3 Materials Science and Engineering Program, State University of New York at Binghamton, Binghamton, New York 13902 USA; 4 Advanced Materials and Processing Branch, NASA Langley Research Center, Hampton, Virginia 23681 USA

## Abstract

The structural stability and mechanical integrity of boron nitride nanotubes (BNNTs) in high temperature environments are of importance in pursuit of their applications that are involved with extreme thermal processing and/or working conditions, but remain not well understood. In this paper, we perform an extensive study of the impacts of high temperature exposure on the structural and mechanical properties of BNNTs with a full structural size spectrum from nano- to micro- to macro-scale by using a variety of *in situ* and *ex situ* material characterization techniques. Atomic force microscopy (AFM) and high resolution transmission electron microscopy measurements reveal that the structures of individual BNNTs can survive at up to 850 °C in air and capture the signs of their structural degradation at 900 °C or above. *In situ* Raman spectroscopy measurements reveal that the BN bonds in BNNT micro-fibrils undergo substantial softening at elevated temperatures of up to 900 °C. The AFM-based nanomechanical compression measurements demonstrate that the mechanical integrity of individual BNNTs remain intact after being thermally baked at up to 850 °C in air. The studies reveal that BNNTs are structurally and mechanically stable materials in high temperature environments, which enables their usages in high temperature applications.

## Introduction

Boron nitride nanotubes (BNNTs) are a type of one-dimensional tubular nanostructure that is composed of hexagonal B-N bond networks^1,2^. As a low density material, BNNTs possess unique structural and physical/chemical properties, many of which are comparable or even superior to their pure carbon counterparts, carbon nanotubes (CNTs)^3^. BNNTs reportedly possess very high Young’s modulus (up to 1.3 GPa) and tensile strength (up to 33 GPa)^4–13^, both of which are comparable to those reported for CNTs. BNNTs also possess piezoelectric and radiation shielding characteristics^14,15^, extraordinary thermal conductivity, and have high thermal and chemical stabilities compared to CNTs^16,17^. For example, BNNTs can survive at up to 800 °C in air, while CNTs start to oxidize at 400 °C^16,18^. The unique light and strong characteristics together with their extraordinary chemical and thermal stability enable BNNTs to excel in high temperature applications, such as reinforcing additives for metal and ceramic nanocomposites that are typically involved with extreme thermal processing and/or working conditions^19,20^. Another unique physical characteristic of BNNTs as reinforcing fillers in metal and ceramic nanocomposites is their insulating property, as B-N bonds have a large bandgap of 5–6 eV^21^. Therefore, BNNTs are stable inside metals, while CNTs may corrode due to their redox potentials^22^. Adding BNNTs does not alter the insulating property of ceramic matrices, while CNTs do. In BNNT-reinforced metal and ceramic nanocomposites, the added BNNTs are typically prepared in dispersed forms and then mixed in with matrix materials either as individual tubes or small bundled fibrils to achieve larger tube-matrix binding areas and thus better property reinforcement. BNNT reinforcement heavily depends on its own structural stability and mechanical integrity under high processing and/or working temperatures. Therefore, a complete understanding of the structural and mechanical properties of BNNTs in high temperature environments is essential to pursue their nanocomposite applications. To date, the studies of the thermal stability of BNNTs remain largely at the bulk or macroscale level, while their properties at individual tube levels (nanoscale) and small-diameter fibrils (microscale) level remain largely unexplored^23–26^. One of the major factors behind the lack of this critical knowledgebase is the low availability of BNNTs in the market due to challenges in sizable synthesis of high quality BNNTs materials. The recent breakthrough in the scalable production of high quality BNNTs has significantly boosted the commercial availability of BNNTs to the research community^27,28^. The so-called HTP (high-temperature pressure) BNNT growth method is reportedly capable of producing highly crystalline, very long, and small diameter BNNTs. Recent studies show that a majority (>97%) of HTP-BNNTs possess one to four walls with outer diameters within the range of 1–6 nm^15^. These small diameter and highly crystalline tubes are ideal for nanocomposites applications.

In this paper, we perform an extensive study of the impacts of high temperature exposure on the structural and mechanical properties of BNNTs with a full structural size spectrum from nano- to micro- to macro-scale by using a variety of *in situ* and *ex situ* material characterization techniques. *In situ* Raman and optical spectroscopy measurements reveal that BNNT micro fibrils can largely survive at up to 1000 °C in air even with substantial thermal-induced BN bond strength weakening. Atomic force microscopy (AFM) and high resolution transmission electron microscopy (HRTEM) measurements reveal that the structures of individual BNNTs can survive at up to 850 °C in air, and also capture the signs of their structural degradation at 900 °C or above. The AFM-based nanomechanical compression measurements demonstrate that the mechanical integrity of individual BNNTs remains intact after being thermally baked at up to 850 °C in air. The findings are useful to better understand the structural stability and mechanical integrity of BNNTs and in pursuit of their applications in high temperature environments.

## Results and Discussion

### *In situ* Raman spectroscopy and optical microscopy characterization of BNNTs

**Figure 1:**
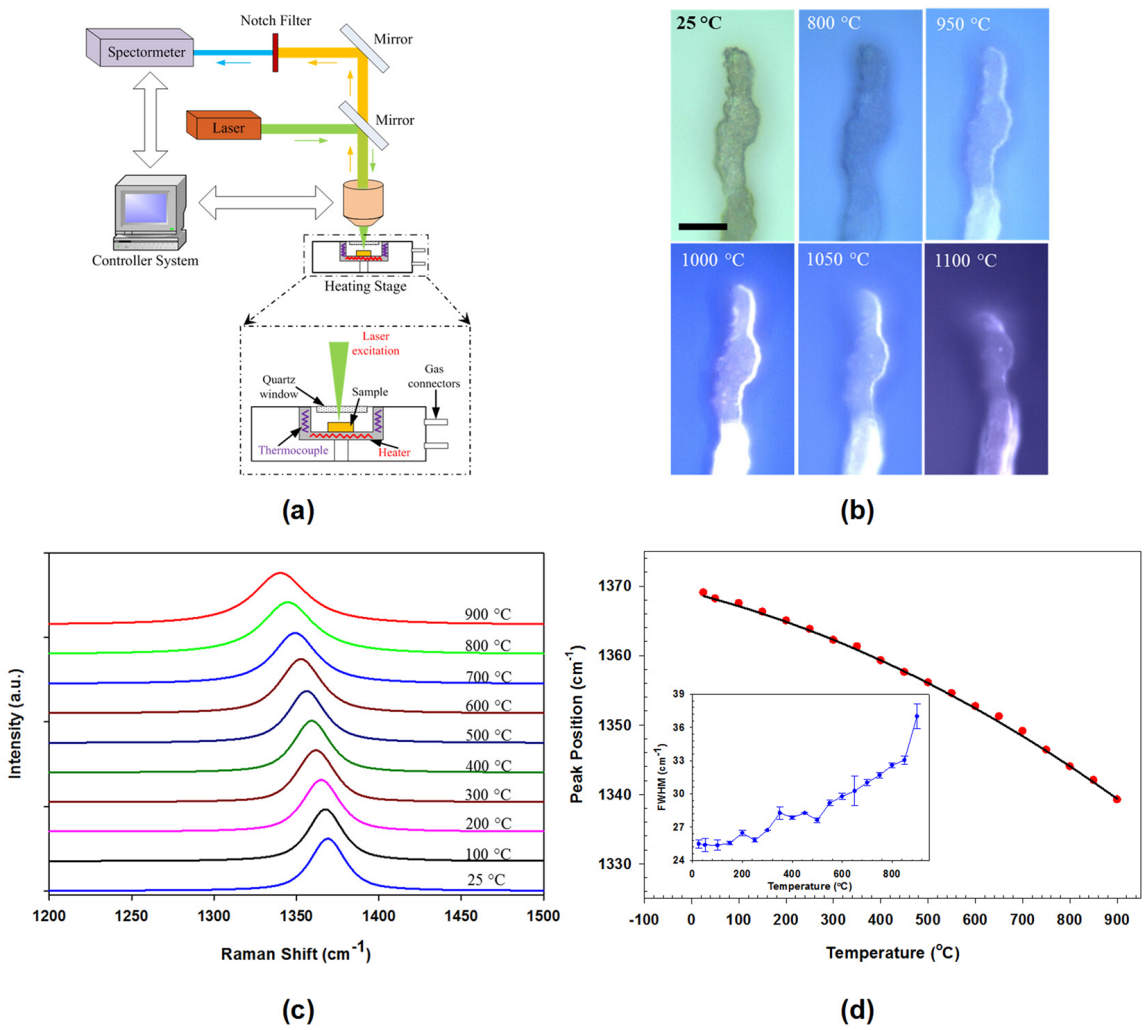
(**a**) A schematic illustration of the experiment setup for *in situ* temperature-dependent Raman spectroscopy and optical microscopy characterization of BNNT microfibrils. (**b**) Selected *in situ* optical images showing the morphology evolutions of one tested BNNT microfibril being heated at different temperatures (the scale bar represents 20 μm). (**c**) Selected *in situ* Raman spectra of the BNNT microfibril shown in (**b**) that were recorded, *in situ*, at different temperatures ranging from 25 °C to 900 °C. (**d**) Temperature dependence of the active G (*E*_*2g*_ mode) peak frequency for the tested BNNT sample. The solid line represents a quadratic polynomial function fitting curve. The inset plot shows the dependence of the corresponding FWHM of the *E*_*2g*_ mode on temperature.

The temperature-dependent structural properties of BNNTs were characterized by using *in situ* Raman spectroscopy and optical microscopy techniques, which are illustrated in the setup shown in Fig. 1(a). BNNT microfibrils, which are exemplified by the tested sample displayed in Fig. 1(b), were pulled directly from as-synthesized BNNTs that were produced using the HTP method. The BNNT microfibril was placed on a computer-controlled heating stage and was illuminated by a laser beam of 785 nm in wavelength. The setup allows the simultaneously *in situ* optical visualization and Raman characterization of the sample at controlled temperatures of up to 1500 °C in air, vacuum or other gas environments. Figure 1(b) shows selected *in situ* optical microscopy snapshots of one BNNT microfibril of about 10–15 µm in lateral dimension under different temperatures. The optical images show that the morphology of the tested BNNT sample remains largely unchanged at up to 950 °C, while noticeable morphological change around the sample’s edge started to occur at 1000 °C, which is ascribed to the oxidation of the BNNTs through reaction with the oxygen in air. The oxidation-induced morphology change was observed to occur in an accelerated mode when the temperature was further increased to 1050 °C and then to 1100 °C. The BNNT sample was largely burned out when the temperature was increased to 1200 °C.

The *in situ* Raman spectroscopic measurements provide microscopic details about the changes of the BN bonds in the tested sample. Figure 1(c) shows the selected *in situ* Raman spectra of the BNNT sample displayed in Fig. 1(b) from room temperature (about 25 °C) to 900 °C (see Methods section for details**)**. The recorded Raman spectra represent the structural characteristics of the B-N bonds in the thermally heated BNNTs that underwent little or no oxidation. At room temperature, BNNTs possess a G band (*E*_2*g*_ mode) peak at 1369 cm^−1 ^^29–31^. The G band peak is found to downshift to about 1339 cm^−1^ at 900 °C, indicating a redshift change of 30 cm^−1^ in wavelength as a result of a temperature increase of about 875 °C. This G band peak is found to gradually downshift with an increasing temperature. Figure 1(d) shows the measured dependence of the G band peak position on the sample temperature. The G band peak position data can be better fitted by using a quadratic polynomial curve that is given as *ω* [*cm*^−1^] = −1.751 × 10^−5^ *T*^*2*^ − 0.01726 *T* + 1369 (*T* is temperature in Celsius), rather than a linear function. The nonlinearity in the observed temperature dependence of the Raman peak position could be ascribed to the anharmonicity that arises as a result of phonon-phonon interactions^32,33^. The downshift of the G band peak can result from the lattice thermal expansion induced-length increase or softening of the BN bond at an elevated temperature. Because the second-order term (nonlinearity) is expected to appear only at high temperatures, the temperature dependence of the G mode frequency shift can be reasonably represented by a linear relation at low temperatures, with its slope being an indicator of the material’s thermal coefficient of redshift. The slope of the curve shown in Fig. 1(d) is in an overall increasing trend. There is little change in the slope of the curve (about −0.024 cm^−1^/°C) at relatively low temperatures of up to 300 °C, followed by an increasing slope segment at up to 600 °C. Then the slope of the curve undergoes little change at up to 900 °C. The overall slope is found to be about −0.028 cm^−1^/°C within the range of 25–600 °C, which is consistent with the thermal coefficient (−0.027 cm^−1^/°C) reported for nanotubes-containing BN soot^34^. In addition to the G mode frequency shift under different temperatures, the full width at half-maximum (FWHM) of the G mode is found to broaden monotonously with rising temperature, as displayed in the inset of Fig. 1(d). The bandwidth increase begins from 100 °C with a nearly linear tendency at a rate of 0.0102 ± 0.0002 cm^−1^/°C for temperatures of up to 850 °C. A more dramatic bandwidth increase is observed at 900 °C. The linear broadening of Raman peaks may be caused by factors such as a temperature increase, oxygen doping, disordering and amorphization^35,36^. The presence of distinct G band Raman peak and the temperature increase-induced gradual redshift and the linear broadening of the Raman peak suggest that the BN lattice structures survive at up to 850 °C in air, which is consistent with the AFM/TEM results that are presented in the next section.

In addition, the FWHM of the G peak at 25 °C is measured to be 25.48 ± 0.34 cm^−1^. It is noted that the linewidth of the G peak quantitatively correlates with the “crystal grain size” of BNNTs due to a wave-vector uncertainty of phonons, and the FWHM is expressed by using *FWHM* = 1417 × 10^−8^/*L* + 8.7 cm^−1^, where *L* is the average crystal grain size^36,37^. The effective crystal grain size in the tested BNNT microfibril sample is calculated to be about 8.4 nm at room temperature, which is close to the average perimeter (9.1 nm) of the HTP-BNNTs that are reported to possess a median diameter of 2.9 nm^38^.

### *Ex situ* AFM and HRTEM imaging characterization of thermally annealed BNNTs

**Figure 2:**
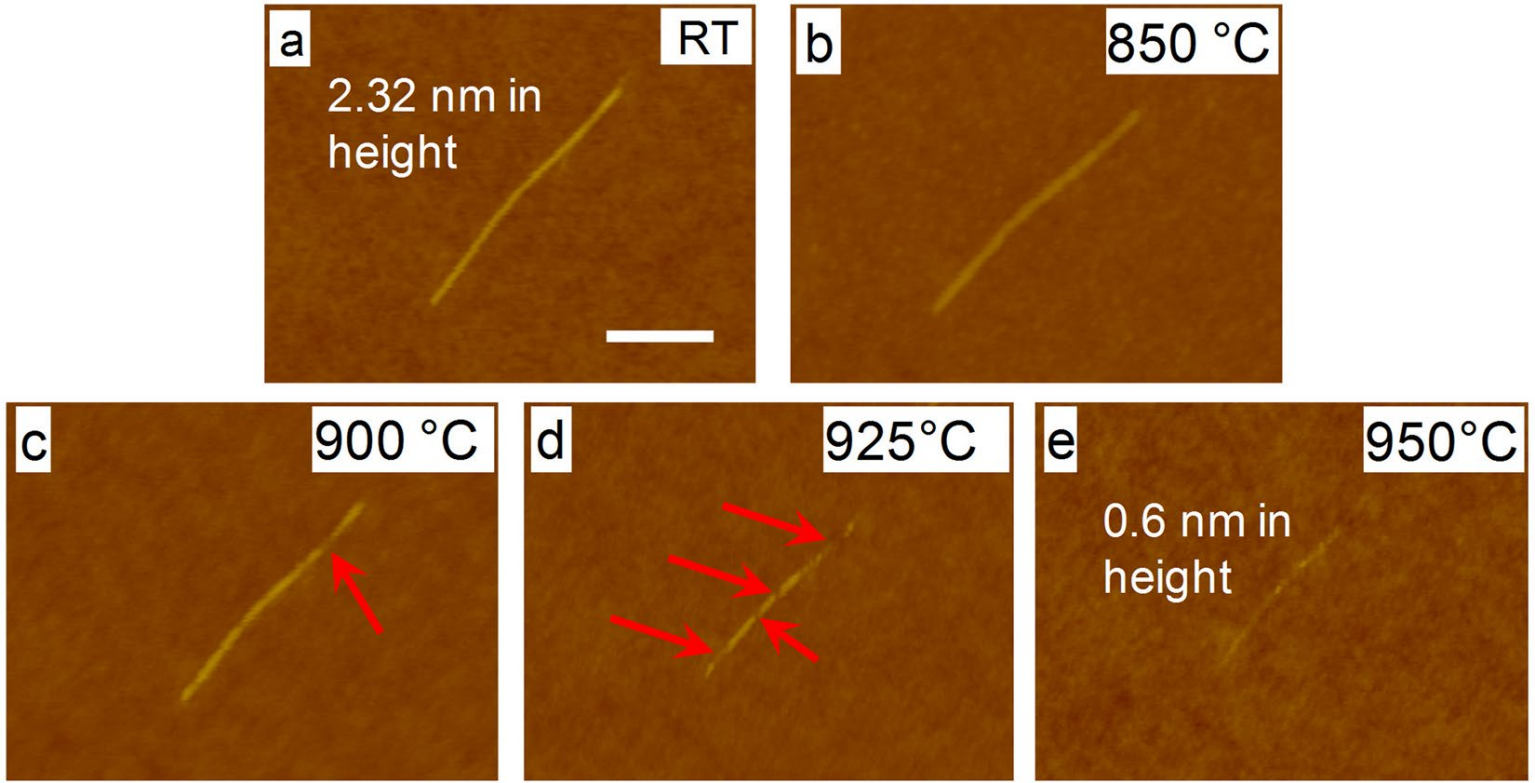
Selected AFM images of one individual BNNT that stayed on a silicon substrate at room temperature (**a**), and after being heated subsequently at 850 °C (**b**), 900 °C (**c**), 925 °C (**d**) and 950 °C (**e**), each for 10 minutes in air. All of the AFM images shown in (**b**–**e**) were taken after the samples were cooled down to room temperature. The scale bar represents 200 nm.

The structural characterization of the microfibrils presented in previous section is useful to understand the overall structural stability and degradation of a microscale BNNT structure in high temperature environments. In this section, we perform nanoscale characterization of thermally annealed BNNTs to understand the thermal oxidation and the structural degradation mode for individual BNNTs by using AFM and HRTEM imaging techniques. Figure 2(a) shows one deposited BNNT of about 2.3 nm in diameter and about 600 nm in length on a flat silicon oxide substrate. The measured tube diameter indicates that it was either a single- or double-walled tube^39,40^. Figure 2(b) shows the same tube after it was first heated to 850 °C and then held for 10 minutes, and cooled down to room temperature afterward, all of which was done in air (see Methods section for details). The AFM measurement shows no sign of structural morphology changes, indicating that the structure of this tube fully survived at this temperature, demonstrating its remarkable resistance to oxidation. The same thermal heating protocol and AFM imaging measurements were repeated on the same tube with temperature increased to 900 °C, 925 °C, and 950 °C, respectively. At 900 °C, the BNNT is found to largely survive with merely a small broken segment of about 30 nm in length as indicated by the arrow in Fig. 2(c). The broken segment is likely caused by the oxidation of the tube segment at that position. The comparison of the images shown in Fig. 2(a) and (c) reveals that the oxidation of the tube did not actually occur from its ends, at which preside atoms with dangling bonds (if the tube ends were not capped), more prone to react with oxygen. The AFM image taken after the sample was heated at 925 °C, which is displayed in Fig. 2(d), clearly reveals a severe structural degradation of the tube having substantial shortening of length and several broken segments. The results show that the oxidation of the tube proceeded in a non-continuous manner along the longitudinal length of the tube, indicating that oxidation may initiate and occur at certain “weak” spots only. Further increasing of the temperature to 950 °C resulted in a severe burnout of the tube with only a small quantity of residues on the substrate, which is shown in Fig. 2(e). In summary, the AFM measurements reveal that the HTP-BNNTs are able to survive at 850 °C in air without any structural degradation, and noticeable structural degradation occurs at 900 °C or above, which is in good agreement with the aforementioned *in situ* Raman measurements as well as thermal gravimetric analysis (TGA) measurements (See Figure S1 in the supplemental materials). The AFM imaging measurement provides useful information about the structural stability and degradation mode of individual BNNTs. However, because the tube was placed on a flat substrate, any possible influence of the substrate on its structural degradation remains an unknown question. We attempted to address this matter by performing HRTEM measurements on individual free-standing BNNTs, which are detailed below.

**Figure 3:**
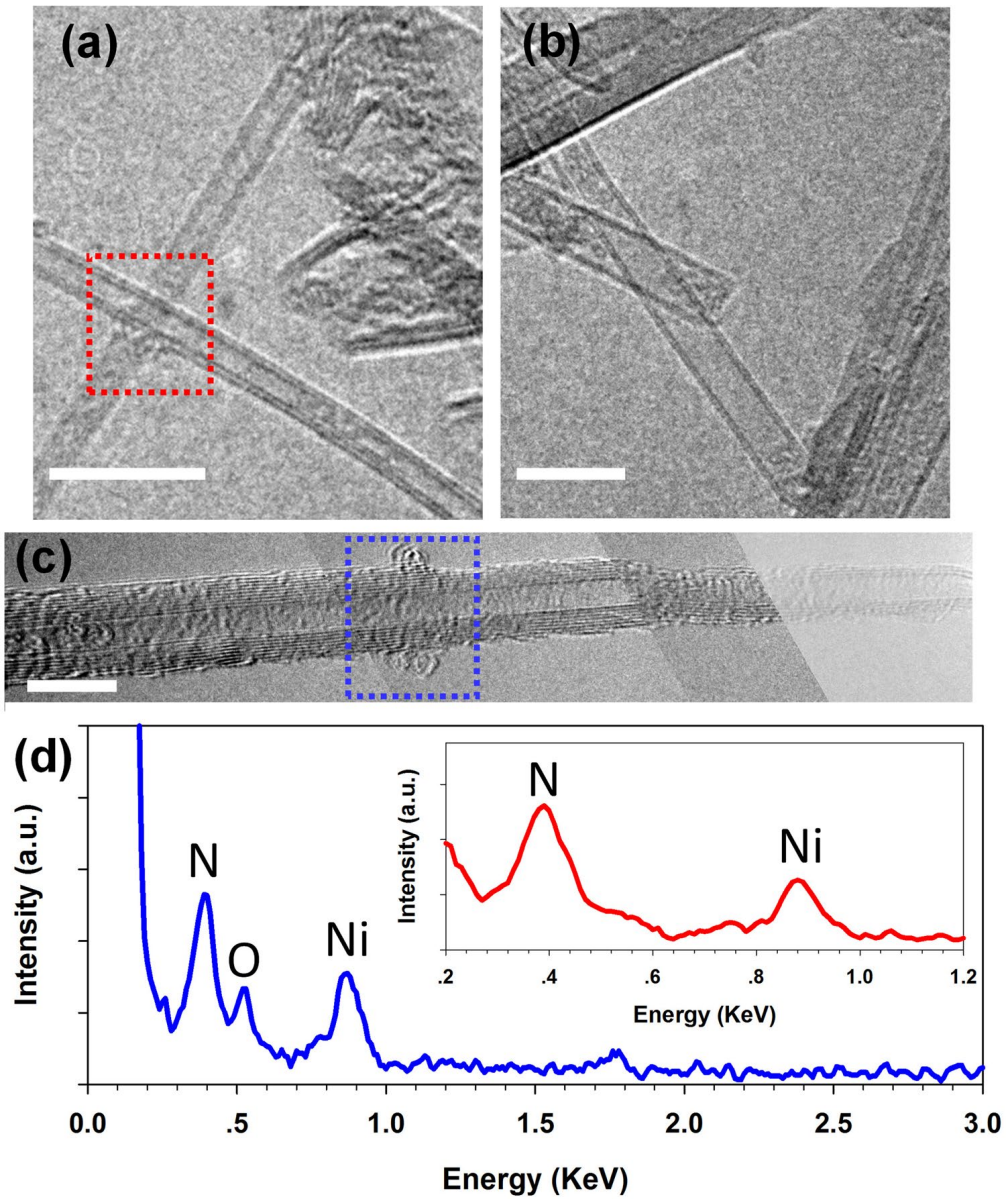
HRTEM images of individual BNNTs deposited on Ni grid after being heated at 850 °C **(a)** and 900 °C **(b**,**c)** in air for 10 mins (the displayed image in (**c**) is an assembled image from a set of HRTEM images that were taken at different portions of the same tube); **(d)** The EDS spectrum for the selected area (blue box) of the BNNT displayed in (**c**). The insert EDS spectrum is for the selected area (red box) of the BNNTs displayed in (**a**). All scale bars represent 10 nm.

**Figure 4:**
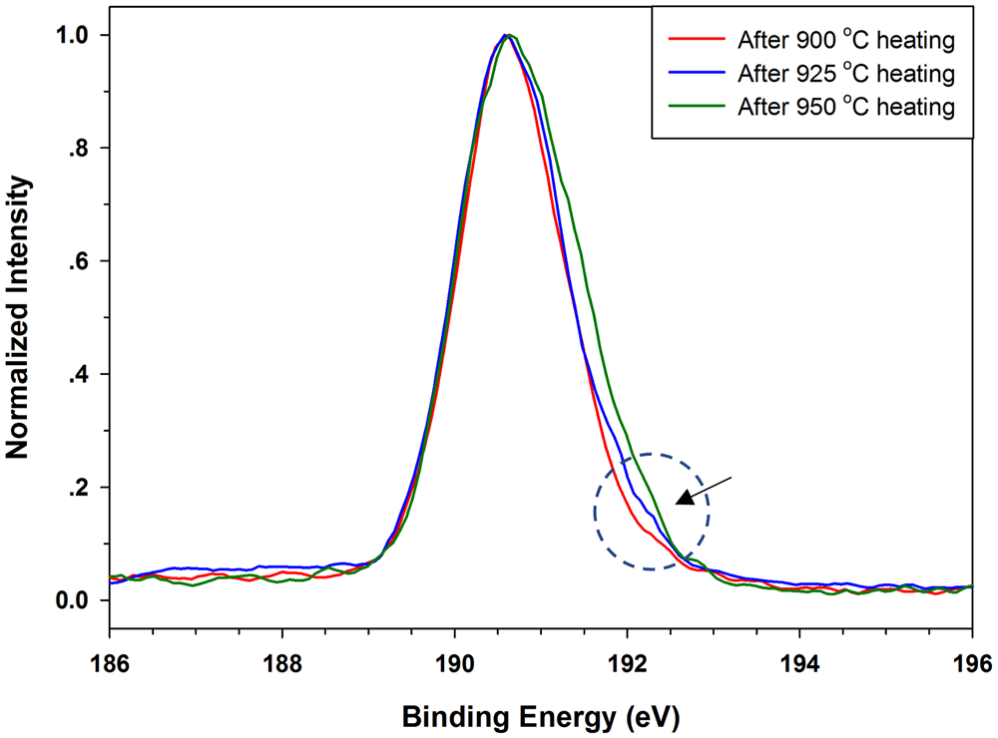
The XPS spectra of a deposited BNNT film after being heated at 900, 925, and 950 °C, respectively, each for 10 minutes in air.

The BNNT samples employed for the HRTEM imaging characterization were from the same batch of dispersed tubes used in the AFM measurements. The dispersed tubes were deposited on Ni TEM grids. The deposited sample was thermally annealed using the same protocol as for those used in the AFM measurements. Figure 3(a) shows two crossing free-standing double-walled BNNTs after being heated at 850 °C for 10 minutes in air. The tube diameters were measured to be 2.1 and 2.3 nm, respectively. Both tubes appear to have quite clean surfaces. The HRTEM measurements clearly show that individual doubled-walled BNNTs can survive 10-minute heating at 850 °C in air. Figure 3(b) shows another two double-walled BNNTs after being heated at 900 °C in air, which also possess clean surface without any noticeable sign of structural degradation. However, the HRTEM inspection of some other tubes baked at 900 °C suggests that oxidation of BNNT might occur at this temperature, and one of the samples is displayed in Fig. 3(c). The displayed tube has a tapered diameter with a decreasing number of walls and diameter from the left to right. It is observed from the image that a cluster of materials was attached to the tube surface at the position where tampering occurred. We performed energy dispersive spectroscopy (EDS) characterization inside the TEM of the selected area (blue box) on the BNNT, and the recorded spectrum is displayed in Fig. 3(d). The EDS spectrum displays three characteristic peaks that are identified to correspond to elements of nitrogen (N), oxygen (O) and nickel (Ni), respectively. By contrast, the insert EDS spectrum in Fig. 3(d) that was recorded for the tubes shown in Fig. 3(a) (red box) displays only the N and Ni peaks. It is believed that the Ni peak came from the interaction of the electron beam with the nickel TEM grid by which the BNNT sample was supported, while the N peak came from the N elements in the BNNT. The existence of the O peak is most likely due to the presence of boron trioxide (B_2_O_3_) residue as a result of the oxidation of BNNTs with oxygen in air around 850–900 °C^14,24^. To confirm the existence of B_2_O_3_ in thermally annealed BNNT samples, we performed *ex situ* x-ray photoelectron spectroscopy (XPS) measurements of BNNT samples that were thermally annealed at 900, 925 and 950 °C in air for 10 minutes, respectively, and the data are displayed in Fig. 4. The displayed peaks of around 190.6 eV in binding energy in the XPS spectra are the characteristics of the BN bond, while the peaks around 192.3 eV are associated with B_2_O_3_^41–44^. The intensity of the peak associated with B_2_O_3_ is found to noticeably increase with temperature. The data indicate that the concentration of B_2_O_3_ in the sample increases with temperature, which is most likely a result of a higher oxidation rate of BN at evaluated temperatures. The presence of B_2_O_3_ is also consistent with a noticeably broader bandwidth of the peak associate with the BN bond as a result of temperature increase from 900 °C to 950 °C. The XPS results provide direct evidence that the oxygen-containing molecules observed on the surface of the BNNT displayed in Fig. 3(c) are likely amorphous B_2_O_3._ When the temperature was increased to 950 °C, no tubes were observed on the TEM grid, indicating that all tubes were either burned out or experienced severe structural damages that might not allow them to remain on the TEM grid. The HRTEM studies show that individual BNNTs can survive at up to 850 °C in air and at least some tubes can survive at up to 900 °C in air. The results are consistent with the finding from the AFM measurements.

### *Ex situ* AFM nanomechanical characterization of thermally annealed BNNTs

**Figure 5:**
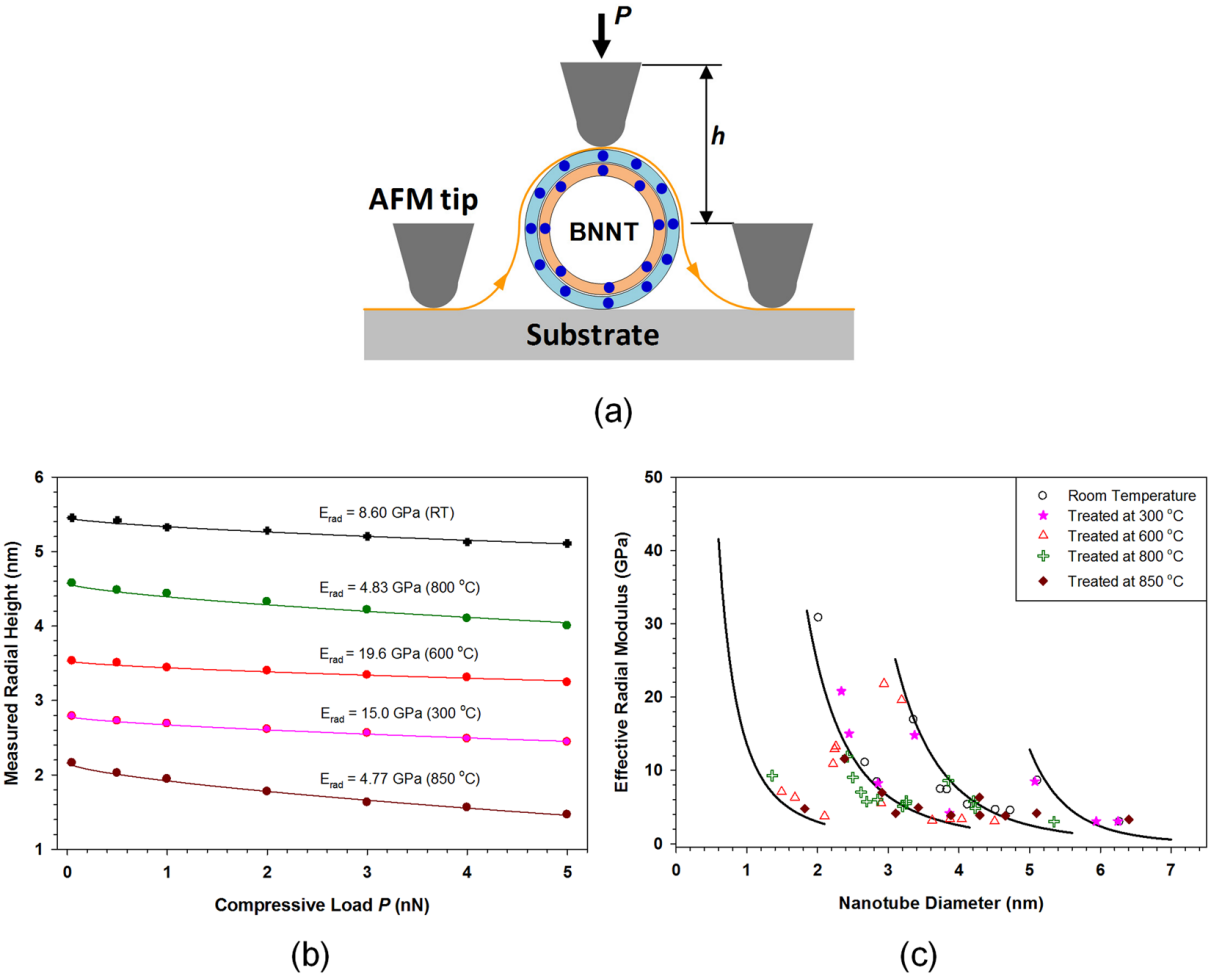
(**a**) A schematic illustration of the AFM-based compression testing scheme on the radial elasticity of an individual BNNT on a flat substrate. (**b**) The recorded compressive load *versus* nanotube height profiles for five selected BNNTs that were thermally annealed at different temperature in air. The dots represent experimental measurements, while the solid curves represent the respective fitting curves based on the Hertzian contact mechanics model. (**c**) The dependence of the measured effective radial modulus of BNNTs on the tube outer diameter at room temperature and after being annealed at 300, 600, 800 and 850 °C, respectively. The solid curves represent the power-function fitting curves of the effective radial elastic moduli data of single- to quadruple-walled BNNTs at room temperature, which are reproduced from ref.^39^.

In this section, we investigate the mechanical property of thermally annealed BNNTs by using AFM-based nanomechanical compression techniques, which are illustrated in Fig. 5(a). The measurements intend to address the question regarding the impact of high-temperature treatments on the tube’s mechanical integrity, which is of great importance in pursuit of their high temperature applications. The AFM-based nanomechanical compression measurement records the transverse height of the tube as a function of the applied normal load and enables the quantification of the effective transverse elastic modulus of individual BNNTs. We have recently employed the same testing technique as illustrated in Fig. 5(a) to investigate the transverse mechanical properties of single- and few-walled HTP-BNNTs at room temperature^39,40^. Our results demonstrate that the effective transverse elastic modulus of individual BNNTs increases with the number of tube walls and decreases with an increase in diameter. The effective transverse elastic modulus can be regarded as a fingerprint representing the structural and mechanical characteristics of nanotubes. Here we employ the same experimental technique as well as the theoretical protocol based on the Hertzian contact model and quantify the effective transverse elastic modulus of individual BNNTs that were thermally annealed at different temperatures in air. Based on the findings of our AFM and HRTEM imaging measurements, the dispersed tubes were thermally annealed at four temperatures of 300, 600, 800 and 850 °C, respectively, at which their structure should remain intact after thermal treatments.

The effective radial modulus of the tested BNNT was quantified from the measured compressive load (*P*) versus cross-section height (*h*, see Fig. 5(a)) using the following Hertzian contact mechanics model^39,40^

1 $$\begin{array}{c}h={h}_{0}-{(\frac{P}{{k}_{1}\sqrt{{h}_{0}}})}^{2/3}-{(\frac{P}{{k}_{2}\sqrt{{[1/{h}_{0}+1/{R}_{tip}]}^{-1}}})}^{2/3}+{(\frac{P}{{k}_{3}\sqrt{{R}_{tip}}})}^{2/3},\\ {k}_{1}=\frac{4}{3}{(\frac{1-{v}_{nt}^{2}}{{E}_{nt}^{rad}}+\frac{1-{v}_{sub}^{2}}{{E}_{sub}})}^{-1},\,{k}_{2}=\frac{4}{3}{(\frac{1-{v}_{tip}^{2}}{{E}_{tip}})}^{-1},\,{k}_{3}=\frac{4}{3}{(\frac{1-{v}_{tip}^{2}}{{E}_{tip}}+\frac{1-{v}_{sub}^{2}}{{E}_{sub}})}^{-1},\end{array}$$

where *E* and *v* represent the elastic modulus (effective radial elastic modulus for BNNTs) and the Poisson’s ratio, respectively, for materials of BNNTs (subscript-*nt*), AFM tips (*tip*) and substrates (*sub*); *h*_0_ represents the original cross-section height of the nanotube on the substrate and is estimated through fitting. The following parameters are employed in the analysis: *E*_*sub*_ = *E*_*tip*_ = 74 GPa; *v*_sub_ = *v*_tip_ = 0.16; *v*_nt_ = 0.2; *R*_*tip*_ = 25 nm^39,40^.

Figure 5(b) shows five selected AFM nanomechanical compression testing profiles for tubes with outer diameters within 1.8 to 5.1 nm. It is noted that the outer diameter of a tube is calculated as *D*_*nt*_ = *h*_0_−*t*, where *h*_0_ represents the original cross-section height of the nanotube on the substrate and *t* = 0.34 nm is the inter-layer distance of the B-N sheet^39^. The tube exposure temperature is marked for each profile and also indicated by the symbol color. All the presented tube-height vs normal load profiles can be very well fitted using the Hertzian contact mechanics model based on eqn. (1), and the effective radial modulus for each tested tube is quantified and marked above the displayed profile in Fig. 5(b). Figure 5(c) shows the dependence of the measured effective radial modulus on the tube diameter. The solid curves are the respective fitting curves to the prior data for single- to quadruple-walled tubes at room temperature (i.e., without any thermal treatment)^39,40^ which are considered as the base curves for the comparison with data for tubes that were thermally annealed at different temperatures. The results show that the obtained data for annealed tubes at all tested temperatures closely follow the respective base curves with reasonable deviations that can be ascribed to the uncertainties in the measurements. The results presented in Fig. 5(c) demonstrate that the mechanical properties of thermally annealed BNNTs, at up to 850 °C in air, remain intact. It is noted that the B-O bond may form due to chemisorption of oxygen molecules on BNNT surfaces at elevated temperatures in air^23^. Our nanomechanical measurement results clearly indicate that the formation of these chemical bonds on BNNT surfaces does not have a material influence on BNNT’s mechanical properties. It is worth mentioning that the structural and mechanical integrity of the BNNTs can survive the melting temperature of aluminum (Al) (660 °C), a basic metal of wide spread usage in the automotive and aerospace industries. The findings strongly support the pursuit of BNNTs for high temperature applications, such as reinforcing fillers for metal and ceramic nanocomposites.

## Conclusion

In this paper, the impacts of high temperature exposure on the structural and mechanical properties of BNNTs were investigated by using a variety of *in situ* and *ex situ* material characterization techniques. *In situ* Raman and optical spectroscopy measurements reveal that BNNT micro fibrils can largely survive at temperatures of up to 1000 °C in air even with substantial thermal-induced BN bond strength weakening. AFM and HRTEM imaging measurements reveal that the structures of individual BNNTs can survive at temperatures of up to 850 °C in air, and also capture the signs of their structural degradation at 900 °C or above. The AFM-based nanomechanical compression measurements demonstrate that the mechanical integrity of individual BNNTs remain intact after being thermally annealed at up to 850 °C in air. The findings are useful to better understand the structural stability and mechanical integrity of BNNTs, especially in the pursuit of their high temperature applications.

## Methods

### Sample preparation and characterization

The BNNTs employed in this study, which were purchased from BNNT LLC, were synthesized using High-Temperature Pressure (HTP) methods and were originally in dry and cotton-like fibrils. The thermal gravimetric analysis (TGA) measurements of BNNTs were performed by using a TA-Q50 (TA Instruments) under a temperature range from room temperature (about 25 °C) to 1000 °C at a rate of 5 °C/min. For AFM, XPS and TEM characterization, BNNTs were first separated/dispersed in deionized (DI) water by ultrasonication with the aid of ionic surfactants. After centrifugation at 2000 rpm, small drops from the top portion of the BNNT solution were deposited on clean Si wafers or nickel grids with lacey support films (Ted Pella, Inc.) and then repeatedly washed using DI water to remove residue surfactants. The BNNT samples were then air-dried at room temperature and subsequently heated up to targeted temperatures (i.e., 850, 900, 925 and 950 °C) in air at a rate of 15 °C/min and held for 10 minutes at each of those final targeted temperatures. All AFM imaging and nanomechanical characterization were performed using a Park Systems XE-70 AFM at room temperature in ambient environment. The employed AFM is equipped with a closed-loop feedback control feature in XYZ axes. Tapping-mode AFM was performed using silicon AFM probes (REFSP-190, Bruker) with a nominal spring constant of 35 N m^−1^. The XPS measurements were performed at room temperature and under ultrahigh vacuum conditions (~10^−8^ torr) using a PHI 5000 VersaProbe instrument (Physical Electronics, Inc.) with monochromatic Al Kα X-rays at 1486.6 eV, a 200 μm diameter spot, and constant pass energy of 23.5 eV at a power of 50 W. The HRTEM and the energy dispersive spectroscopy (EDS) characterization were performed using a JEM 2100 F TEM (JEOL Ltd) operated at accelerating voltages of 120–200 kV.

### *In situ* temperature-dependent Raman spectra measurements

For Raman spectra measurements, the BNNT microfibril was placed on a flat quartz substrate within a programmable temperature controlled stage (Linkam TS 1500), in which the BNNTs sample temperature can be controlled in a range from room temperature (about 25 °C) to 1500 °C. The Raman spectra were obtained using Renishaw InVia Confocal Raman microscope with a 785 nm wavelength excitation laser and a nominal power of 300 mW. The samples were heated stepwise up to 1100 °C in air at a rate of 15 °C/min and held 10 minutes for every 50 °C mark to stabilize the temperature of the sample. In order to prevent/minimize laser heating induced sample damage or additional Raman shift, line scans of laser beams were used to illuminate the sample, instead of focusing laser beams on any particular spots.

### *Ex situ* temperature-dependent effective radial elastic modulus measurements

In the BNNT radial elasticity measurements, dispersed nanotubes were first deposited on a flat silicon oxide substrate at room temperature. The deposited nanotubes were thermally heated using the same approach as employed in the *in situ* Raman measurement to specific temperatures that range from the room temperature to 850 °C, and then cooled down to room temperature for radial elasticity measurements using AFM-based compression tests that are illustrated in Fig. 5(a). The details of the measurements can be found in refs^39,40^. In brief, an AFM tip was controlled to scan an individual BNNT on the substrate in contact mode at a specified compressive load with the scanning direction perpendicular to the tube axis. The employed AFM scanning rate was 50 nm s^−1^. Silicon AFM cantilevers (CSG 01, NT-MDT) with nominal spring constants of 0.003–0.13 N m^−1^ were employed. The actual spring constant of each employed CSG 01 AFM cantilever was calibrated using the thermal tuning method and found to be in the range of 0.03–0.11 N m^−1^.

### Data Availability

The datasets generated during and/or analysed during the current study are available from the corresponding author on reasonable request.

## Electronic supplementary material

Supporting Information
