# Author Correction: Quantitative Characterization of Structural and Mechanical Properties of Boron Nitride Nanotubes in High Temperature Environments

**DOI:** 10.1038/s41598-018-23081-3

**Published:** 2018-03-14

**Authors:** Xiaoming Chen, Christopher M. Dmuchowski, Cheol Park, Catharine C. Fay, Changhong Ke

**Affiliations:** 1 Micro- and Nanotechnology Research Center, State Key Laboratory for Manufacturing Systems Engineering, Xi’an Jiaotong University, Xi’an, Shaanxi 710049 China; 2 Department of Mechanical Engineering, State University of New York at Binghamton, Binghamton, New York, 13902 USA; 3 Materials Science and Engineering Program, State University of New York at Binghamton, Binghamton, New York, 13902 USA; 4 Advanced Materials and Processing Branch, NASA Langley Research Center, Hampton, Virginia 23681 USA

Correction to: *Scientific Reports* 10.1038/s41598-017-11795-9, published online 12 September 2017

This Article contains a typographical error in the Introduction:

“BNNTs reportedly possess very high Young’s modulus (up to 1.3 GPa).”

should read:

“BNNTs reportedly possess very high Young’s modulus (up to 1.3 TPa)”

