# Supplementary material for: Quantitative Characterization of Structural and Mechanical Properties of Boron Nitride Nanotubes in High Temperature Environments
Source: Sci Rep. 2017 Sep 12;7:11388. doi: 10.1038/s41598-017-11795-9 (PMC5595806; doi:10.1038/s41598-017-11795-9)
Supplement: Supplementary file 1 — Supporting Information [file 41598_2017_11795_MOESM1_ESM.pdf]

Supporting Information

Quantitative Characterization of Structural and Mechanical Properties of Boron Nitride Nanotubes in High Temperature Environments

Xiaoming Chen,[1,2] Christopher M Dmuchowski,[2,3] Cheol Park,[4] Catharine C. Fay,[4] and Changhong Ke[2,3*]

[1]Micro- and Nanotechnology Research Center, State Key Laboratory for Manufacturing Systems Engineering, Xi'an Jiaotong University, Xi'an, Shaanxi 710049, China

[2]Department of Mechanical Engineering, State University of New York at Binghamton, Binghamton, New York 13902, USA

[3]Materials Science and Engineering Program, State University of New York at Binghamton, Binghamton, New York 13902, USA

[4]Advanced Materials and Processing Branch, NASA Langley Research Center, Hampton, Virginia 23681, USA

*To whom correspondence should be addressed. cke@binghamton.edu

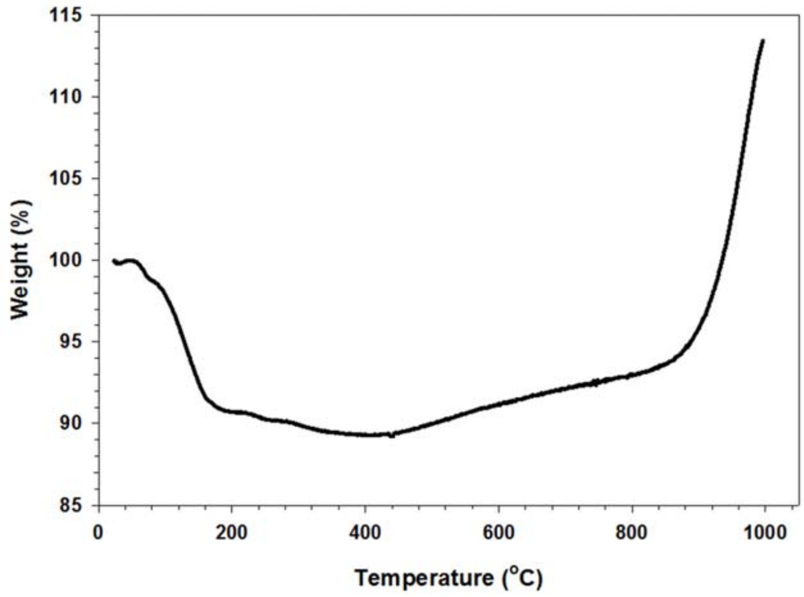

Figure S1: The dependence of the weight change of a BNNT sample on temperature during heating in TGA at a rate of 5 ˚C/min in air. The increase segment from about 440˚C to about 850˚C is attributed to the oxidation of the residual boron in as-synthesized BNNTs. The increase segment from about 850˚C to 1000˚C is an indication of the oxidation of BNNTs.
